# Predicting the risk of HIV infection among internal migrant MSM in China: An optimal model based on three variable selection methods

**DOI:** 10.3389/fpubh.2022.1015699

**Published:** 2022-10-25

**Authors:** Shangbin Liu, Danni Xia, Yuxuan Wang, Huifang Xu, Lulu Xu, Dong Yuan, Ajuan Liang, Ruijie Chang, Rongxi Wang, Yujie Liu, Hui Chen, Fan Hu, Yong Cai, Ying Wang

**Affiliations:** ^1^School of Public Health, Shanghai Jiao Tong University School of Medicine, Shanghai, China; ^2^Shanghai Municipal Center for Disease Control and Prevention, Shanghai, China; ^3^Renji Hospital, Affiliated With the School of Medicine Shanghai Jiao Tong University, Shanghai, China

**Keywords:** prediction model, nomogram, internal migrant MSM, HIV, random forests, stepwise selection, univariable selection

## Abstract

**Introduction:**

Internal migrant Men who have sex with men (IMMSM), which has the dual identity of MSM and floating population, should be more concerned among the vulnerable groups for HIV in society. Establishing appropriate prediction models to assess the risk of HIV infection among IMMSM is of great significance to against HIV infection and transmission.

**Methods:**

HIV and syphilis infection were detected using rapid test kits, and other 30 variables were collected among IMMSM through questionnaire. Taking HIV infection status as the dependent variable, three methods were used to screen predictors and three prediction models were developed respectively. The Hosmer-Lemeshow test was performed to verify the fit of the models, and the net classification improvement and integrated discrimination improvement were used to compare these models to determine the optimal model. Based on the optimal model, a prediction nomogram was developed as an instrument to assess the risk of HIV infection among IMMSM. To quantify the predictive ability of the nomogram, the C-index measurement was performed, and internal validation was performed using bootstrap method. The receiver operating characteristic (ROC) curve, calibration plot and dynamic component analysis (DCA) were respectively performed to assess the efficacy, accuracy and clinical utility of the prediction nomogram.

**Results:**

In this study, 12.52% IMMSMs were tested HIV-positive and 8.0% IMMSMs were tested syphilis-positive. Model A, model B, and model C fitted well, and model B was the optimal model. A nomogram was developed based on the model B. The C-index of the nomogram was 0.757 (95% CI: 0.701–0.812), and the C-index of internal verification was 0.705.

**Conclusions:**

The model established by stepwise selection methods incorporating 11 risk factors (age, education, marriage, monthly income, verbal violence, syphilis, score of CUSS, score of RSES, score of ULS, score of ES and score of DS) was the optimal model that achieved the best predictive power. The risk nomogram based on the optimal model had relatively good efficacy, accuracy and clinical utility in identifying internal migrant MSM at high-risk for HIV infection, which is helpful for developing targeted intervention for them.

## Introduction

In recent years, men who have sex with men (MSM) have emerged as a high-risk group for HIV infection in China, with the rate of HIV infection rising year by year (1). A large-scale systematic analysis reported that the national HIV prevalence among MSM from 2001 to 2018 was 5.7%, and the study prevalence ranged from 0 to 22.91% (2). Previous studies have shown that the internal migrant MSM (IMMSM) constituted most of the MSM populations, ranging from 71.2 to 91.3% (3–5). IMMSM is defined as changes of usual residence within countries and have had sex with a male in the last 6 months (6). Migration within China has been identified as a major factor influencing the transmission of HIV in China, and the high migration rate among MSM populations poses a huge challenge to HIV prevention and control (1, 7).

Because of the better employment opportunities, less social discrimination, and more comfortable living environment, a large number of MSM moved to the metropolis and developed regions, but due to the imperfection of China's household registration system and urban social security system, the migrant populations had lower access to social benefits such as education and health services than the local populations (1, 8). According to prior studies, migrant MSM had lower levels of HIV knowledge and fewer opportunities for HIV testing compared with the local MSM populations, but they were at higher risk of engaging in high-risk sexual behaviors, such as unprotected anal intercourse (UAI), multiple sexual partners (MSP), and transactional sex (9–11). It has been documented that the HIV infection rates in migrant MSM were higher than those in local MSM (1, 12). Migrant MSM, which has the dual identity of MSM and floating population, should be more concerned among the vulnerable groups in society.

Joint United Nations Programme on HIV/AIDS (UNAIDS) has set the 95-95-95 strategy to achieve the goal of ending the global HIV epidemic by 2030, with the goal of making 95% of people living with HIV aware of their HIV serostatus by 2030 (13). Nevertheless, HIV testing rate among MSM has been low in China. A study including 988 participants found that only 57.1% of MSM have been tested for HIV in the past year (14). Thus, other methods are required to distinguish those at high-risk for HIV infection. Establishing appropriate prediction models to assess the risk of HIV infection in migrant MSM populations is beneficial for an individual to aware risk of infection and proactively avoid adverse factors, and is also in favor of the government to identify high-risk groups and integrate limited resources for targeted interventions specific to this population. While several HIV risk assessment tools have been targeted at MSM domestic and overseas (15–18), none has yet been developed for internal migrant MSM.

A variety of risk factors associated with HIV infection among MSM have been identified within the existing literatures. Among these factors, sociodemographic characteristics like age and education, behaviors such as UAI, sex trade and alcohol use before sex, sexually transmitted diseases have been widely studied (19–23). However, most studies to date have overlooked the important role that psychological factors play on HIV infection. As a sexual minority, MSM lives in surroundings full of stressors, such as stigma, prejudice and discrimination, which may compound negative mental health. Accordingly, compared with the heterosexual communities, MSM have suffered from serious mental health problems, including depression, distress, and trauma (24). Unfortunately, psychological problems and the relationship between these problems and HIV infection among migrant MSM is an underexplored area. Therefore, prediction models that incorporating psychological factors may assess the vulnerabilities for HIV infection more effectively.

Given these multiple gaps in knowledge of risk assessments of HIV infection among migrant MSM, in the present study, we explored numerous risk factors including sociodemographic characteristics, behaviors and experiences, condom use related factors, STDs infection status and psychological variables for HIV infection. After that, three different methods (Boruta, stepwise selection and univariable selection) were used to establish and validate prediction models. Then, an optimal model was selected to develop nomogram as an instrument to assess the risk of HIV infection among IMMSM, which may help to develop targeted interventions for them in the future.

## Method

### Study design and sampling

A cross-sectional study was performed simultaneously in four cities in China: east China (Shanghai), southern China (Shenzhen), northeastern China (Shenyang) and Southwest China (Kunming). It is difficult to target MSM by using probability sampling because they tend to hide their sexual orientation. The “snowball” sampling technique, which is advantageous in addressing population covertness was chosen for the present study. In each city, with the help of non-government organizations or local center for disease control and prevention, 5–10 socially adept MSM were recruited as “seeds” for the “snowball sampling”. Subsequently, with the introduction of the initial “seeds”, more and more MSM were recruited and acted as new “seeds”. Each participant took part in a face-to-face interview with a trained staff in which they were informed of the details of the study. Written informed consent was obtained from participants before the survey. The recruitment procedure and the design of this study were approved by the Ethics Committee of School of Public Health, Shanghai Jiao Tong University.

### Inclusion and exclusion criteria

Inclusion criteria: physiologically male, at least 18 years of age, and self-reported to have had sex with a male in the past 6 months. Exclusion criteria: those whose current place of residence and registered place are the same; cannot complete the questionnaire independently. 229, 195, 92, and 187 IMMSMs were recruited in Shanghai, Shenyang, Kunming and Shenzhen, respectively. All of them completed the test and questionnaire and were included in this study. All participants were given one free HIV test as well as a $17.84 subsidy.

### Measurements

HIV and syphilis infections were tested by using rapid test kits (Beijing Aipu Medical Equipment Co.). The information collected through the questionnaire included basic demographic information (age, marriage, monthly income, etc.), homosexual sexuality (condom use, commercial sex, etc.), substance use (alcohol, cigarettes, drugs).

#### Rosenberg self-esteem scale (RSES)

The level of self-esteem was assessed by the 10 items, 4-point Likert-type RSES. Answers ranging from 1 (strongly disagree) to 4 (strongly agree), the higher the scores, the higher the level of self-esteem.

#### University of California at Los Angels loneliness scale (ULS)

Loneliness was assessed by ULS, which included 8 items, answers ranging from 1 (never) to 4 (often). The higher total scores indicated the stronger feeling of loneliness.

#### Patient health questionnaire-9 (PHQ-9)

Depression was assessed by the PHQ-9, with the total scores from 0 to 27. Answers range from 0 (never) to 3 (almost everyday). The higher scores indicated the higher level of depression.

#### Entrapment scale (ES)

Entrapment was assessed by the 16-item ES with answers ranging from 0 (never) to 4 (always), the higher the scores, the higher the level of entrapment.

#### Defeat scale (DS)

Defeat was assessed by the 16-item DS, with answers ranging from 0 (never) to 4 (always), the higher the scores, the higher the level of defeat.

#### Interpersonal needs questionnaire (INQ-15)

Interpersonal need was measured using the 15-item, 7-point Likert-type INQ-15. Answers were rated ranging from 1 (strongly not comply) to 7 (strongly comply) where a higher total score indicates a higher level of unmet interpersonal needs.

#### Suicidal ability scale (SAS)

The 9-item Likert-type SAS was applied to measure individual's suicidal ability. Answers were rated ranging from 0 (strongly disagree) to 8 (strongly agree), with a higher total score indicating higher suicidal ability.

#### Social support scale (SSS)

We employed the SSS to assess subjectively recognized social support from family, friends, and significant others. The scale is a 7-point Likert scale with 12 items, ranging from 1 (very strongly disagree) to 7 (very strongly agree). A higher total score indicated higher social support.

#### Sexual compulsivity scale (SCS)

The 10-item Likert-type SCS was applied to measure individual's out-of-control sexual thoughts and behaviors. Answers were rated ranging from 1 (strongly disagree) to 4 (strongly agree), with a higher total score indicating higher sexual compulsivity.

All of the above scales are widely used in the MSM population (25–40), and total of 31 independent variables were collected.

### Statistical analysis

#### Scale reliability test

Scale reliability is broadly defined in the population as the ratio of the variance of true scores to the variance of observed scores, while Cronbach's coefficient alpha is a statistic for assessing scale reliability based on internal consistency (41). Cronbach's coefficient alpha ≥0.7 represents a good correlation and alpha < 0.5 represents an unacceptable correlation (42, 43).

#### Feature description

Categorical variables were expressed as frequencies and percentages, and continuous variables were described as medians and quartiles (non-normal distributions). Chi-square test, corrected Chi-square test and Fisher exact test were used to assess the differences in categorical variables between HIV-positive MSM and HIV-negative MSM. Differences in continuous variables between HIV-positive MSM and HIV-negative MSM were evaluated using Mann–Whitney U test. All tests were two-sided and *P* ≤ 0.05 was set as the level of significant difference.

#### Variable selection methods

##### Boruta

Boruta is based on the same idea which forms the foundation of the random forest classifier, namely, that by adding randomness to the system and collecting results from the ensemble of randomized samples one can reduce the misleading impact of random fluctuations and correlations. The Boruta algorithm consists of following steps (44):

1. Extend the Information System by Adding Copies of all the 31 Variables.
2. The Added Attributes Are Processed to Remove Their Association With the Response.
3. Run a Random Forest Classifier on the Extended Information System and Gather the Z Scores Computed.
4. Find the Maximum Z Score Among Shadow Attributes (MZSA), and Then Assign a hit to Each Attribute That Scored Better Than MZSA.
5. For Each Attribute With Undetermined Importance Perform a two-Sided Test of Equality With the MZSA.
6. Deem the Attributes Which Have Importance Significantly Lower Than MZSA as ‘Unimportant' and Permanently Remove Them From the Information System.
7. Deem the Attributes Which Have Importance Significantly Higher Than MZSA as ‘Important'.
8. Remove all Shadow Attributes.
9. Repeat the Procedure Until the Importance Is Assigned for all the Attributes, or the Algorithm Has Reached the Previously set Limit of the Random Forest Runs.

##### Stepwise selection

Stepwise selection is a variation of forward selection. At each step of the variable selection process, after a variable has been added to the model, variables are allowed to be removed from the model (45). For example, if the significance of a given predictor is higher than a specific threshold, it is removed from the model. The iterative process is ended when the pre-defined stopping rule is satisfied.

##### Univariate selection

The steps for univariate selection were: variables with *P* ≤ 0.1 in the comparison of differences between HIV-positive and HIV-negative groups were included in the univariate logistic regression, and the variables with *P* ≤ 0.05 in the univariate logistic regression would be used to develop the prediction model.

#### Hosmer-Lemeshow test

Assessing the goodness of fit of a model is an important and essential part of any modeling exercise. The Hosmer-Lemeshow (H-L) test for the goodness of fit of logistic regression is very popular because it is easy to implement, simple to interpret, and widely adopted by popular statistical packages (46). It is widely used for the evaluation of risk-scoring models in medicine (47, 48). The null hypothesis of H-L test is that there is no significant difference between the predicted and observed values, which implies a perfect fit of the model (49).

#### Determination of the optimal model

Three prediction models were developed by using multivariate logistic regression and we compared the models by determining the net reclassification improvement (NRI) and integrated discrimination improvement (IDI) to select the optimal model.

#### Development and validation of nomogram

The nomogram is a visualization of the prediction model. In this study, a nomogram will be developed based on the best prediction model to assess the risk of HIV infection among IMMSM. The receiver operating characteristic (ROC) curve, calibration plot and dynamic component analysis (DCA) were respectively performed to assess the efficacy, accuracy and clinical utility of the prediction nomogram. Discrimination was calculated using the C-index, ranging from 0.5 to 1.0 (1.0 means perfect discrimination) (50), and internal validation was performed using bootstrap method. Bootstrap samples are random samples drawn with replacement from the original sample, and 1,000 bootstrap samples were fitted in this study to calculate a relatively corrected C-index.

## Results

### Statistical description

Cronbach's alpha of mental health scales: RSES was 0.842, ULS was 0.824, PHQ-9 was 0.905, ES was 0.969, DS was 0.938, INQ-15 was 0.854, SAS was 0.787, SSS was 0.949, SCS was 0.934. The results showed good reliability for all mental health scales.

703 IMMSM were included in this study. The median age of the IMMSMs was 31.00 (26.00, 35.00) years old, 576 (81.9) IMMSMs were unmarried, 442 (62.9%) IMMSMs had received a college education, 88 (12.52%) IMMSMs were tested HIV-positive, 56 (8.0%) IMMSMs were tested syphilis-positive, 134 (19.1%) IMMSMs had experienced verbal violence due to their sexual orientations, 350 (49.8%) IMMSMs had irregular homosexual anal sex partners, 368 (52.3%) IMMSMs had substance use experience. Specific demographic and scale scores are shown in Table 1.

**Table 1:** Characteristics of the IMMSMs.

**No**.	**Characteristics**	**Total (*n* = 703)**	**HIV-negative (*n* = 615)**	**HIV-positive (*n* = 88)**	**p-value**
1	Age	31.00 (26.00, 35.00)	31.00 (26.00, 35.00)	32.00 (28.00, 36.00)	0.143
2	Score of CUAS	24.00 (22.00, 28.00)	25.00 (22.00, 28.00)	24.00 (20.00, 26.25)	0.003
3	Score of CUSS	24.00 (20.00, 29.00)	25.00 (21.00, 29.00)	23.00 (17.00, 27.00)	0.001
4	Score of CUSNSS	16.00 (14.00, 20.00)	16.00 (14.00, 20.00)	16.00 (13.00, 19.00)	0.080
5	Score of CUSES	26.00 (22.00, 29.00)	26.00 (22.00, 29.00)	24.00 (20.00, 28.00)	0.048
6	Score of RSES	19.00 (16.00, 22.00)	19.00 (16.00, 22.00)	18.00 (15.00, 22.25)	0.224
7	Score of ULS	17.00 (13.00, 21.00)	17.00 (13.00, 21.00)	16.00 (12.00, 20.00)	0.075
8	Score of PHQ-9	7.00 (3.00, 9.50)	7.00 (3.00, 9.50)	6.00 (2.00, 9.25)	0.146
9	Score of ES	11.00 (1.00, 24.00)	10.00 (2.00, 24.00)	12.00 (0.00, 25.25)	0.478
10	Score of DS	18.00 (11.00, 28.00)	18.00 (11.00, 28.00)	15.50 (12.00, 25.25)	0.278
11	Score of INQ-15	42.00 (30.00, 52.00)	42.00 (30.00, 52.00)	42.50 (28.00, 51.00)	0.770
12	Score of SAS	20.00 (12.00, 28.00)	21.00 (12.00, 28.00)	20.00 (6.50, 26.25)	0.101
13	Score of SSS	61.00 (48.00, 72.00)	60.00 (48.00, 72.00)	61.00 (49.00, 72.00)	0.744
14	Score of SCS	22.00 (18.00, 26.00)	22.00 (18.00, 26.00)	20.00 (15.75, 25.00)	0.101
15	HIV education				0.783
	NO	306 (43.5)	266 (43.3)	40 (45.5)	
	YES	397(56.5)	346(56.7)	48(54.5)	
16	VCT				0.632
	YES	553(78.7)	486(79.0)	67(76.1)	
	NO	150 (21.3)	129 (21.0)	21 (23.9)	
17	Verbal violence				0.170
	YES	134(19.1)	112(18.2)	22(25.0)	
	NO	569 (80.9)	503 (81.8)	66 (75.0)	
18	Physical violence				0.506
	YES	41(5.8)	34(5.5)	7(0.8)	
	NO	662 (94.2)	581 (94.5)	81 (92.0)	
19	Regular homosexual anal sex partners				0.538
	YES	305(43.4)	270(43.9)	35(39.8)	
	NO	398 (56.6)	345 (56.1)	53 (60.2)	
20	Irregular homosexual anal sex partners				0.598
	YES	350 (49.8)	309 (50.2)	41 (46.6)	
	NO	353(50.2)	306(49.8)	47(53.4)	
21	Homosexual sex trade				0.787
	YES	32 (4.6)	27 (4.4)	5 (5.7)	
	NO	671 (95.4)	588 (95.6)	83 (94.3)	
22	Substance use				0.051
	YES	368 (52.3)	331 (53.8)	37 (42.0)	
	NO	335 (47.7)	284 (46.2)	46 (58)	
23	PrEP				0.996
	YES	30 (4.3)	26 (4.2)	4 (4.5)	
	NO	673 (95.7)	589 (95.8)	84 (95.5)	
24	Syphilis				0.021
	YES	56 (8.0)	43 (7.0)	13 (14.8)	
	NO	647 (92.0)	572 (93.0)	75 (85.2)	
25	Education				< 0.001
	Primary school and below	11 (1.5)	7 (1.1)	4 (4.5)	
	Junior high school	109 (15.5)	84 (13.7)	25 (28.5)	
	High school	141 (20.1)	118 (19.2)	23 (26.1)	
	College and above	442 (62.9)	406 (66.0)	36 (40.9)	
26	Marriage				0.626
	Unmarried	576 (81.9)	501 (81.5)	75 (85.3)	
	Married	95 (13.5)	86 (14.0)	9 (10.2)	
	divorced	32 (4.6)	28 (4.5)	4 (4.5)	
27	Monthly income ($)				< 0.001
	≤ 446.40	182 (25.9)	167 (27.1)	15 (17.0)	
	446.55–892.80	240 (34.1)	193 (31.4)	47 (53.4)	
	892.95–1785.60	196 (27.9)	175 (28.5)	21 (23.9)	
	≥1785.75	85 (12.1)	80 (13.0)	5 (5.7)	
28	Time for residence				0.497
	1 year and below	177 (25.2)	158 (25.7)	19 (21.6)	
	1–5 years	296 (42.1)	254 (41.3)	42 (47.7)	
	5 years and above	230 (32.7)	203 (33.0)	27 (30.7)	
29	Sexual orientation				0.361
	Heterosexuality	12 (1.7)	11 (1.8)	1 (1.1)	
	Homosexuality	467 (66.4)	408 (66.3)	59 (67.0)	
	Bisexual	198 (28.2)	176 (28.6)	22 (25.0)	
	Unclear	26 (3.7)	20 (3.3)	6 (6.9)	
30	Smoking				0.170
	Never	471 (67.0)	421 (68.5)	50 (56.8)	
	Sometimes	101 (14.4)	85 (13.8)	16 (18.2)	
	Always	48 (6.8)	39 (6.3)	9 (10.2)	
	Everyday	83 (11.8)	70 (11.4)	13 (14.8)	
31	Drinking before sex				0.489
	Never	480 (68.3)	424 (68.9)	56 (63.6)	
	Sometimes	206 (29.3)	178 (28.9)	28 (31.9)	
	Always	13 (1.8)	10 (1.6)	3 (3.4)	
	Every time	4 (0.6)	3 (0.6)	1 (1.1)	

Values are presented as median (IQR) or number (%).

Variable Description: No. 1, Age, Age of the participant at the time of the survey; No. 2, CUAS, the Condom Use Attitude Scale, with higher scores indicating more positive attitudes; No. 3, CUSS, the Condom Use Skills Scale, assessed subjects' negotiation skills with condom use and refusal of unprotected sex, the higher the scores, the better the condom use skills; No. 4, CUSNSS, the Condom Use Subjective Normative Scale, assessed others' (family, friends, peers at the same job, significant others) perceptions of participants' adherence to condom use during anal sex, with higher scores indicating more positive attitudes; No. 5, CUSES, the Condom Use Self-Efficacy Scale, assessed their ability to participate effectively in condom use, and the higher the scores, the higher the condom use self-efficacy; No. 6, RSES, the Rosenberg Self-Esteem Scale, assessed their level of self-esteem, and the higher the scores, the higher the level of self-esteem; No. 7, ULS, the UCLA (University of California at Los Angels) Loneliness Scale, assessed their level of loneliness, the higher the scores, the stronger the loneliness; No. 8, PHQ-9, the Patient Health Questionnaire, assessed their level of depression, and the higher the scores, the higher the level of depression; No. 9, ES, the Entrapment Scale, assessed their level of entrapment, and the higher the scores, the higher the level of entrapment; No. 10, DS, the Defeat Scale, assessed their level of defeat, and the higher the scores, the higher the level of defeat; No. 11, INQ-15, the Interpersonal Needs Questionnaire, assessed their level of interpersonal needs, the higher the scores, the more unmet interpersonal needs; No. 12, SAS, the Suicidal Ability Scale, assessed their level of suicidal ability, and the higher the scores, the higher the level of suicidal ability; No. 13, SSS, the Social Support Scale, assessed their level of social support, the higher the scores, the higher the support; No. 14, SCS, the Sexual Compulsivity Scale, assessed their level of sexual compulsivity, the higher the scores, the stronger the compulsivity; No. 15, experienced of receiving HIV education, Yes or No; No. 16, had HIV Voluntary Counseling and Test, Yes or No; No. 17, experienced of verbal violence such as harassment, insults, threats, Yes or No; No. 18, experienced of physical violence such as pushing, Yes or No; No. 19, had regular homosexual anal sex partners, Yes or No; No. 20, had irregular homosexual anal sex partners, Yes or No; No. 21, supplied sexual services for male sexual partners, Yes or No; No. 22, used substances, Yes or No; No. 23, had taken pre-exposure prophylaxis in the past, Yes or No; No. 24, had infected with syphilis, Yes or No; No. 25, education level; No. 26, marriage status; No. 27, Monthly income; No. 28, residence time; No. 29, sexual orientation; No. 30, frequency of smoking, never means not even once, sometimes means 3 to 5 times a month, always means more than 3 times a week, and Everyday means at least once a day; No. 31, frequencies of drinking behavior before sex.

### Screening of predictive factors by three methods

Three HIV infection prediction models were developed based on three different variable screening methods. The Boruta algorithm screened 11 factors (Education, score of CUAS, score of CUSS, score of RSES, score of ULS, score of PHQ-9, score of ES, score of DS, score of INQ-15, score of SAS and score of SCS) and developed model A (Supplementary Table 1). Stepwise selection method (both forward and backward) was used to screen 11 factors (Age, Education, Marriage, Monthly income, Verbal Violence, Syphilis, score of CUSS, score of RSES, score of ULS, score of ES and score of DS) and built model B (Supplementary Table 2). There were 9 variables with *P*-values ≤ 0.1 in the exploration of differences between HIV-positive IMMSM and HIV-negative IMMSM (Table 1). Subsequently, univariate logistic regression was created for each of these variables with HIV infection status, and the results showed that 7 variables (score of CUAS, score of CUSS, score of ULS, Education, Monthly income, Syphilis and Substance use) had *P*-values ≤ 0.05 (Supplementary Table 3). So, the univariate selection screened 7 factors and built model C. The coefficients and *P*-values of the variables for each model were shown in Table 2. The results of Hosmer-Lemeshow test showed that the corresponding *P*-values of the 3 models were 0.7124, 0.1066, and 0.153, respectively. The *P*-values of the three models were greater than 0.05, indicating that these models had good fits and were valid.

**Table 2:** Models established by 3 variable selection methods.

**Variables**	**Estimate**	**z-value**	**P-value**	**OR (95% CI)**
**Model A**				
Score of CUAS	−0.053	−1.533	0.125	0.949(0.887–1.015)
Score of CUSS	−0.043	−1.988	0.047	0.958(0.919–1.000)
Score of RSES	−0.042	−1.167	0.243	0.959(0.893–1.028)
Score of ULS	−0.045	−1.300	0.194	0.956(0.894–1.022)
Score of PHQ-9	−0.026	−0.736	0.461	0.974(0.908–1.043)
Score of ES	0.037	2.155	0.031	1.038(1.003–1.074)
Score of DS	−0.029	−1.217	0.224	0.972(0.927–1.017)
Score of INQ-15	−0.012	−0.932	0.351	0.988(0.962–1.013)
Score of SSS	−0.001	−0.115	0.908	0.999(0.979–1.019)
Score of SCS	−0.024	−1.101	0.271	0.977(0.936–1.019)
**Education**				
Primary school and below	-	-	-	1
Junior high school	−0.355	−0.506	0.613	0.701(0.183–3.037)
High school	−0.663	−0.937	0.349	0.515(0.133–2.261)
College and above	−1.417	−2.036	0.042	0.243(0.064–1.044)
**Model B**				
Age	0.030	1.630	0.103	1.031(0.993–1.069)
Verbal Violence	−0.429	−1.453	0.146	0.651(0.370–1.180)
Syphilis	0.746	2.019	0.043	2.108(0.991–4.254)
score of CUSS	−0.054	−2.620	0.009	0.947(0.910–0.987)
score of RSES	−0.054	−1.504	0.132	0.947(0.882–1.016)
score of ULS	−0.061	−1.908	0.056	0.941(0.883–1.001)
score of ES	0.031	1.843	0.065	1.032(0.998–1.066)
score of DS	−0.048	−2.037	0.042	0.953(0.909–0.998)
**Education**				
Primary school and below	-	-	-	1
Junior high school	−0.622	−0.840	0.401	0.537(0.129–2.501)
High school	−0.897	−1.207	0.227	0.408(0.098–1.904)
College and above	−1.443	−1.995	0.046	0.236(0.059–1.066)
**Marriage**				
Unmarried	-	-	-	1
Married	−1.034	−2.309	0.021	0.355(0.14–0.821)
divorced	−0.667	−1.064	0.287	0.513(0.131–1.615)
**Monthly income**				
≤ 446.40	-	-	-	1
446.55-892.80	0.849	2.527	0.012	2.338(1.233–4.64)
892.95-1785.60	0.366	0.962	0.336	1.442(0.688–3.087)
≥1785.75$	−0.646	−1.081	0.280	0.524(0.146–1.584)
**Model C**				
Score of CUAS	−0.035	−1.044	0.296	0.965(0.904–1.032)
Score of CUSS	−0.061	−2.181	0.029	0.941(0.89–0.994)
Score of CUSES	0.022	0.73	0.465	1.023(0.964–1.088)
Syphilis	0.699	1.944	0.052	2.011(0.963–3.977)
Substance use	−0.328	−1.29	0.197	0.721(0.436–1.184)
**Education**				
Primary school and below				1
Junior high school	−0.9	−1.26	0.208	0.407(0.103–1.795)
High school	−1.148	−1.609	0.108	0.317(0.08–1.399)
College and above	−1.589	−2.312	0.021	0.204(0.055–0.862)
**Monthly income ($)**				
≤ 446.40				1
446.55-892.80	0.938	2.805	0.005	2.555(1.354–5.062)
892.95-1785.60	0.482	1.28	0.201	1.62(0.780–3.448)
≥1785.75$	−0.161	−0.28	0.779	0.851(0.250–2.478)

### Hosmer-Lemeshow test for model fit

Model A, model B and model C were performed H-L test respectively, and the *P*-values of the results were 0.683, 0.663, and 0.579 respectively. All results were insignificant, which indicated that all three models were fitted well.

### Comparison of models and determination of the best prediction model

Table 3 shows the NRI and IDI between these three models. Taking model A as the reference, we compared model A with model B. The values of NRI and IDI were greater than zero and both were significant (*P* < 0.05), which indicates that model B was superior to model A. The values of NRI and IDI for model C compared to model A were not statistically significant, which indicates model C did not exhibit superiority for predicting HIV infection among IMMSM. Taking model B as the reference, model C compared with model B, the values of NRI and IDI were less than zero and both were significant (*P* < 0.05), which indicates that model B was superior to model C. There was a significant improvement in model B compared to model A and model C. Therefore, model B was the optimal prediction model to predict the risk of HIV infection among IMMSM in this study.

**Table 3:** Comparison of the prediction ability among different models through NRI and IDI.

**Variable**	**Model A–B**	**Model A–C**	**Model B–C**
NRI	0.466	0.197	−0.362
2.5%CI	0.252	−0.026	−0.579
97.5%CI	0.680	0.419	−0.145
P-value	< 0.001	0.083	0.001
IDI	0.036	0.005	−0.031
2.5%CI	0.016	−0.015	−0.050
97.5%CI	0.056	0.026	−0.011
P-value	< 0.001	0.611	0.002

### Development and validation of the nomograms

**Figure 1:**
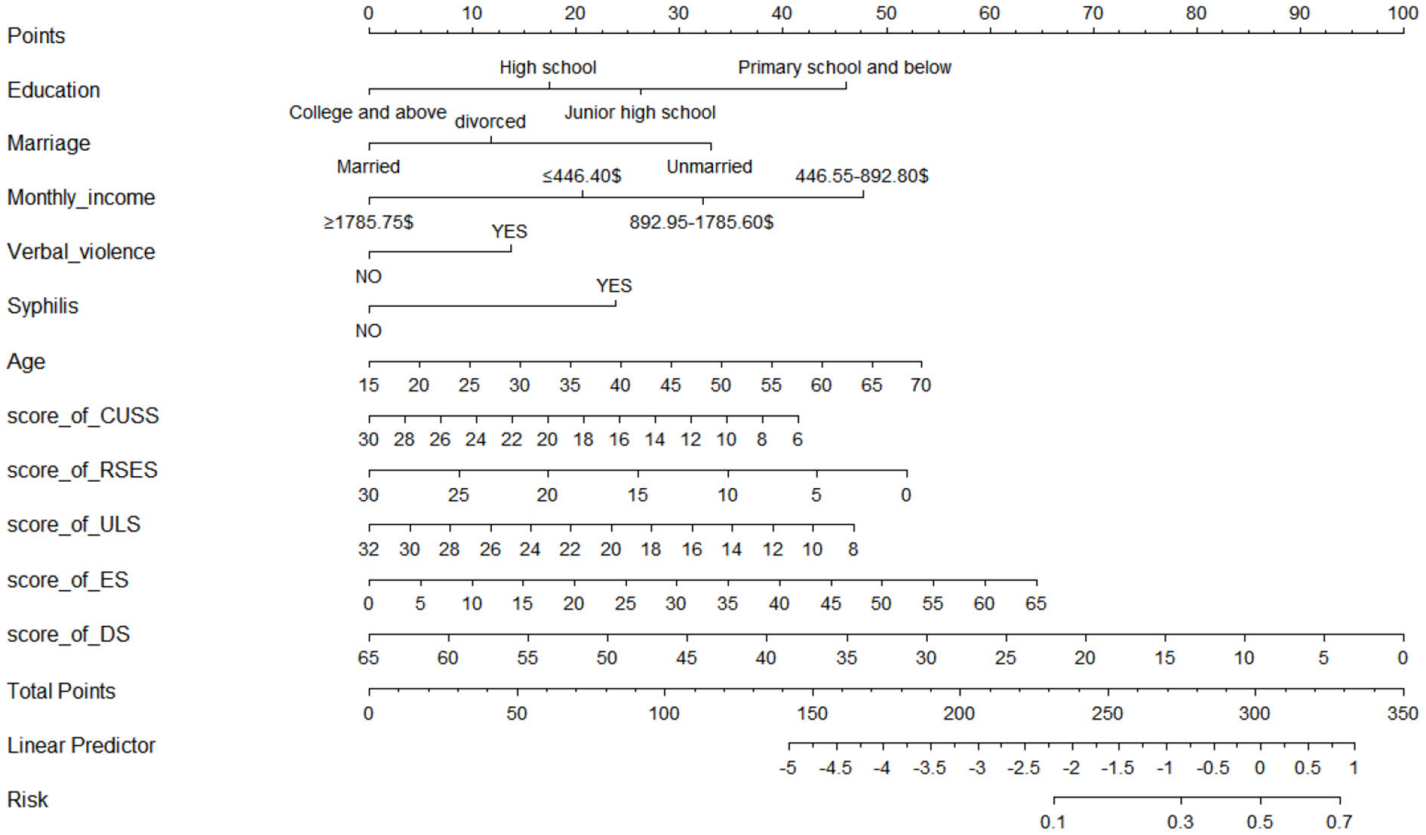
The nomogram for predicting HIV infection among internal migrant MSM.

**Figure 2:**
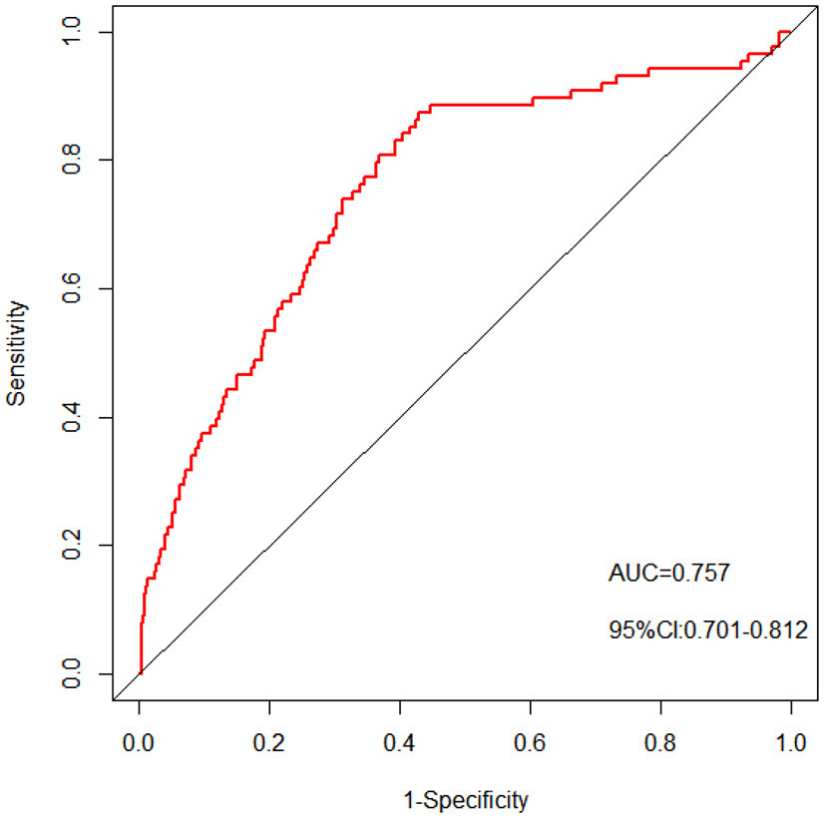
The pooled AUC of the ROC curve. The y-axis means the true positive rate of the risk prediction. The x-axis means the false positive rate of the risk prediction. The red line represents the performance of the nomogram.

**Figure 3:**
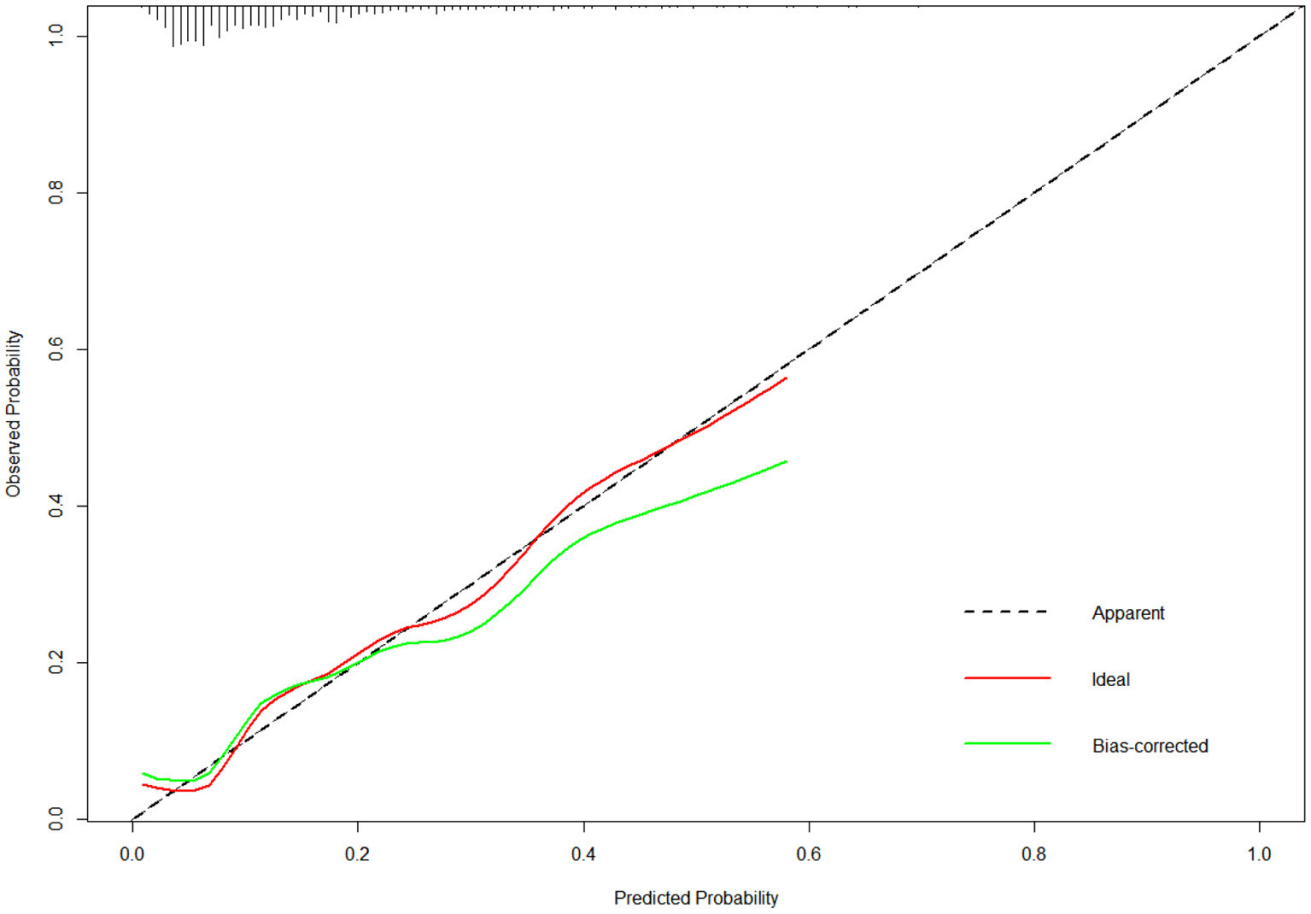
The calibration curves of the nomogram. The x-axis represents the predicted risk of HIV infection. The y-axis represents the actual diagnosed HIV incidence. The diagonal dotted line represents a perfect prediction by an ideal model. The green line represents the performance of the nomogram, of which a closer fit to the diagonal dotted line represents a better prediction.

**Figure 4:**
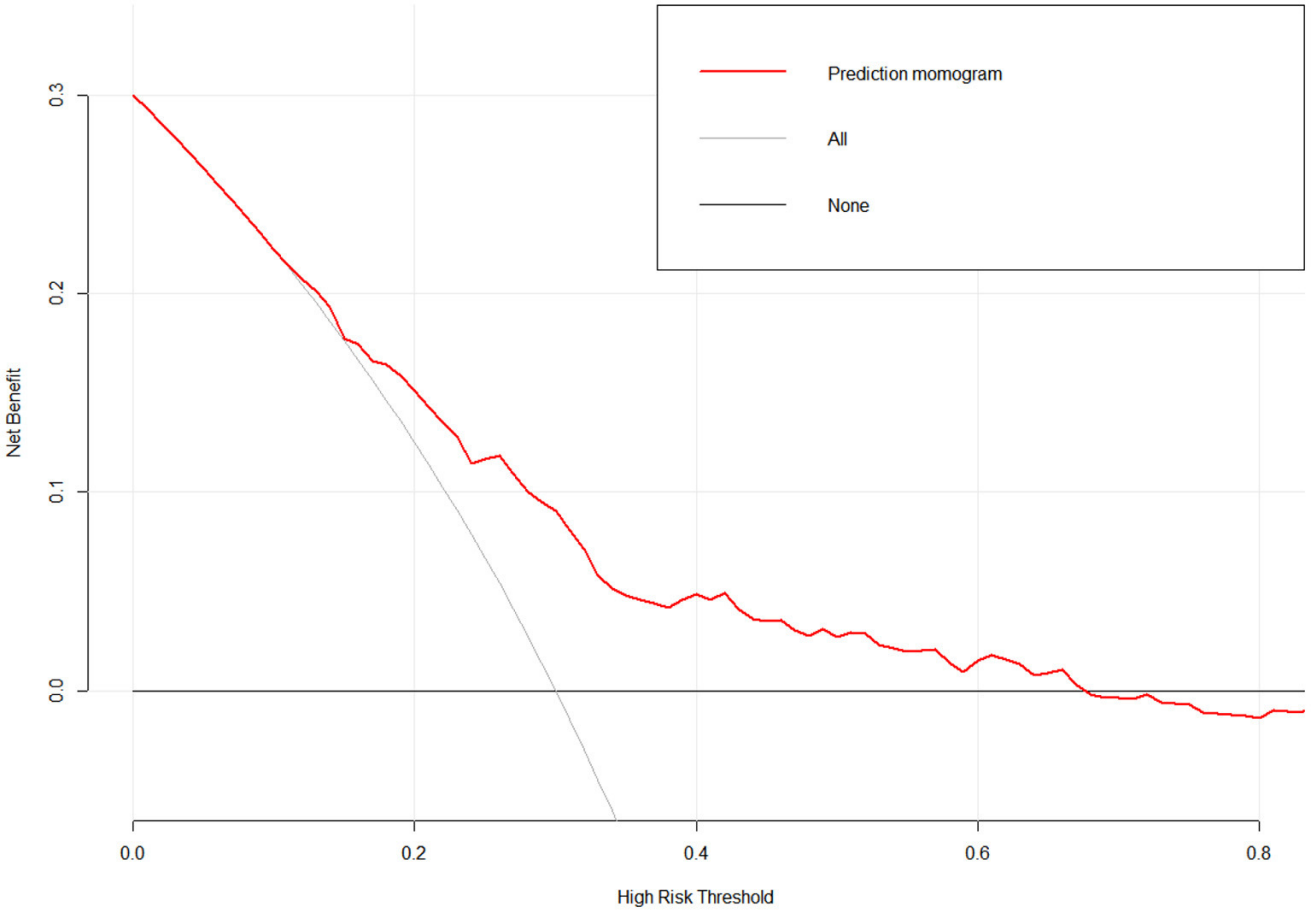
Decision curves of the nomogram. The y-axis measures the net benefit. The red line represents the HIV incidence risk nomogram. The light black solid line represents the assumption that all patients are diagnosed as DN. Deep black solid line represents the assumption that no patients are diagnosed as HIV.

A nomogram with 11 variables (Age, Education, Marriage, Monthly income, Verbal Violence, Syphilis, score of CUSS, score of RSES, score of ULS, score of ES and score of DS) was constructed based on the model B (Figure 1), and a dynamic nomogram can be accessed and used via a URL: https://liubi.shinyapps.io/DynNomapp/. The AUC of the nomogram was 0.757(95% CI: 0.701–0.812), and the ROC showed in Figure 2. Calibration curves of the nomogram demonstrated good consistency between the predicted and observed results (Figure 3). The result of DCA revealed that using the nomograms to predict HIV infection among IMMSM added more net benefit than the treat-all or treat-none strategies (Figure 4), suggesting good clinical utility of the nomograms. The C-index for the prediction nomogram was 0.757 (95% CI: 0.701–0.812), which was confirmed to be 0.705 via bootstrapping validation.

## Discussion

In this study, 30 variables were collected among IMMSM through questionnaire on four aspects—sociodemographic characteristics, homosexual behavior, substance use, and mental health. Another 2 variables-HIV and syphilis infection were detected using rapid test kits. Taking HIV infection status as the dependent variable, three methods (Boruta, stepwise selection and univariable selection) were used to screen predictors and three prediction models (model A, model B and model C) were developed by multivariate logistic regression respectively. By comparing NRI and IDI between these three models, model B was considered the optimal model. The nomogram passed the validation of ROC, calibration curve and DCA, indicating its good efficacy, accuracy and clinical utility in predicting HIV infection among IMMSM. The C-index of internal verification was 0.705, which indicated that the model had medium prediction accuracy. To summarize the above validation results, the nomogram of the model has moderate predictive power.

### Sociodemographic characteristics in the prediction nomogram

Four sociodemographic characteristics (age, education level, monthly income and marital status) were incorporated in our prediction nomogram. Age was found to be a risk factor for HIV infection among IMMSM, as participants with higher age were more likely to report HIV infection. Several studies have reported similar results (2, 51, 52). One reason might be that older IMMSM have been exposed to HIV for a longer time. It has been argued that as IMMSM get older, they might have a greater chance of becoming infected over time, so prevalence within older age will be higher than that in the younger groups (52). Additionally, unprotected anal intercourse is more common in older MSM than in younger MSM (53). Another possible reason was that the older MSM may not be as prudent as the younger counterparts when engaging in sexual behavior due to their rich sexual experience.

In the prediction nomogram, lower levels of education contributed higher risk values for HIV infection among IMMSM. In the one hand, the less educated IMMSM usually have less chance to obtain health information and support (2), which may lead to their poorer abilities on protecting themselves from HIV infection. In the other hand, ADDIN EN.CITE (54, 55) some studies found that condom use frequencies as well as the number of consistent sexual partner were significantly higher among MSM with higher levels of education (e.g., college students or teachers) than among others (56–58), which suggested that low HIV infection risk in more educated IMMSM may benefits from their low participation in risky sexual behaviors.

In this study, the monthly income range with the highest contribution risk value was 446.55–892.80$, then 892.95–1785.60$, followed by ≤ 446.40$, and the lowest was ≥1,785.75$. Socioeconomic status (SES) is an important determinant of health. It seems complicated to explain the contribution of monthly income to the risk value of HIV infection among IMMSM. In terms of MSM with higher economic status, they were more willing to participate in HIV testing and counseling services, possibly because they were more capable of affording the cost of these services (20, 58, 59). What's more, individual with higher incomes tend to have better educational background and have better access to health information. As for MSM with low incomes (60, 61), it was reported by two studies conducted in China that they were more likely to take pre-exposure prophylaxis, which is an efficient way to prevent HIV infection. Additionally, MSM with lower incomes were less likely to report UAI (62). ADDIN EN.CITE (63) ADDIN EN.CITE (64, 65) our findings suggest that the mechanism how individual's economic status play a role on their HIV infection risk among IMMSM is warranted in the future studies.

In China, some MSM would marry women due to widespread social rejection and pressure from their parents (66). Being married to a woman does not completely preclude them from having sex with men (37, 67), but they might reduce the frequency of homosexual sex to avoid disclosure of sexual orientation. In addition, the responsibilities of maintaining a family consumed so much time and energy that they were unable to engage in homosexual behavior as they once did. The vast majority of IMMSM in this study were unmarried, which is consistent with most studies (68–70). Unmarried IMMSM might be more assertive about their sexual orientation and take advantage of their migrant status to escape the discrimination and pressure from society and family. They also changed their homosexual partners and engaged in risky sexual behaviors more frequently (71, 72).

### Psychometric measurements in the prediction nomogram

The lower score of RSES contributed to the higher risk value. Self-esteem serves as a protective buffer against negative emotions that arise from disruptive or threatening experiences. For MSM, self-esteem acted as a sexual risk buffer for MSM (73). But the widespread stigmatization and marginalization of MSM could continue destroying their self-esteem. On the one hand, MSM with low self-esteem tended to engage in less self-care behaviors and was more likely to be involved in high-risk sexual behaviors (74, 75). On the other hand, the desire for self-esteem drove them to seek validation and stronger social relationships among homosexual partners, which might lead to more sexual partners and more frequent high-risk sexual behavior (76, 77).

In the prediction nomogram, lower ULS scores contributed to higher risk values for HIV infection. Higher scores of ULS indicated stronger feelings of loneliness. Loneliness has been defined as the distress that exists between actual and desired relationships (78, 79). For internal migrant MSM, what they expect was to build a good social relationship with other MSM (80). Higher scores of ULS indicated higher levels of loneliness and a greater likelihood of being in poor social relationships, which may lead to fewer homosexual partners and less risky sexual behaviors, thus reducing the risk of HIV infection.

Gilbert and Allen defined entrapment as a personal feeling that an individual was in an unfavorable state or situation and had a strong motivation to escape or get rid of the stressor, but was not capable to escape (81). Compulsive sexual behavior, sexual abuse, promiscuity and commercial sexuality are common among MSM and can increase the risk of HIV infection (82–85). Internal migrant MSM with higher ES scores might be trapped by these behaviors and unable to escape. ADDIN EN.CITE (86–89).

Defeat can arise from a loss or reduction in one's perceived ability to compete for social status (81, 90). For internal migrant MSM, defeat might stem from being rejected due to sexual orientation, feeling inferior to others, or feeling incompetent in various roles. Defeat involved a loss of emotional autonomy and a sense of being broken, expressed through statements such as “I feel like a loser” (91). To cope with the defeat, they might reduce their exploration and involvement in homosexual relationships, and thus indirectly reduce the risk of HIV infection (86, 92).

### Behavior, experiences and STDs in the prediction nomogram

MSM were often subjected to verbal violence because their sexual orientation was not accepted by mainstream society (93–95). Prolonged exposure to verbal violence might have a negative impact on their behavior and mental health. Experiences of verbal violence were reported to be associated with high-risk sexual behaviors and substance use which were also risk factors for HIV infection (96–98). In addition, verbal violence might indirectly increase the risk of HIV infection by promoting the occurrence of anxiety and depression (99–101).

It is well known that condoms are one of the most effective HIV prevention methods, and condom use skills can directly affect MSM's consistent use of condoms (102–104). Higher scores of CUSS represent stronger condom use negotiation skills and unprotected sex refusal skills (39, 105). These skills can help MSM protect themselves from HIV infection by promoting them to use condoms consistently when they engage in high-risk sexual behaviors.

Syphilis is another highly prevalent sexually transmitted disease among MSM and it shares common risk factors with HIV (106–108). If an IMMSM was infected with syphilis, it means that he might also has some risk factors for HIV infection. More importantly, syphilis infection can directly increase the risk of HIV infection. Strong epidemiologic studies have provided substantial evidence that syphilis infection, one of the causes of genital ulcer disease, facilitates HIV transmission (109). Syphilitic ulcers disrupt epithelium and mucosa, which provides a portal of entry for HIV virus (110).

## Conclusion

Our study screened the risk factors related to HIV infection among IMMSM through three variable-selection methods (Boruta, stepwise selection and univariable selection). The model established by stepwise selection methods incorporating 11 risk factors (age, education, marriage, monthly income, verbal violence, syphilis, score of CUSS, score of RSES, score of ULS, score of ES and score of DS) was the optimal model that achieved the best predictive power by comparing the NRI and IDI between three models. The risk nomogram based on the optimal model had relatively good efficacy, accuracy and clinical utility in identifying IMMSM at high-risk for HIV infection, which is helpful for developing targeted intervention for them.

## Limitations

Some limitations of the study should be noted. First, snowball sampling was used to recruit participants, and those who participated in the study might share some similar characteristics. Second, many of the scales and questions were made into questionnaires that were filled out subjectively by the participants, which made the objectivity of the study insufficient. Finally, the entire study was cross-sectional based, and results need to be further confirmed by cohort studies.

## Data availability statement

The raw data supporting the conclusions of this article will be made available by the authors, without undue reservation.

## Ethics statement

The studies involving human participants were reviewed and approved by the Ethics Committee of School of Public Health, Shanghai Jiao Tong University School of Medicine. The patients/participants provided their written informed consent to participate in this study.

## Author contributions

YC and YiW: designed the study. SL, DY, and LX: designed and completed the questionnaire production. RC and RW: created the research database and entered research data. AL: assisted in data collection. SL, YL, and HC: completed the statistical analysis of the data. SL and DX: wrote the first draft of the article. FH, YC, and YiW: have revised the first draft into the final draft. All authors contributed to the article and approved the submitted version.

## Funding

This study was supported by Shanghai Jiao Tong University School of Public Health Local High-level University Achievement-oriented Top-notch Cultivation Programme for Undergraduate Students (Grant No. 18ZYGW20), the National Natural Science Foundation of China (Grant No. 71603166), and the fifth round of Shanghai public health three-year action plan key disciplines construction (Grant No. GWV-10.1-XK18).

## Conflict of interest

The authors declare that the research was conducted in the absence of any commercial or financial relationships that could be construed as a potential conflict of interest.

## Publisher's note

All claims expressed in this article are solely those of the authors and do not necessarily represent those of their affiliated organizations, or those of the publisher, the editors and the reviewers. Any product that may be evaluated in this article, or claim that may be made by its manufacturer, is not guaranteed or endorsed by the publisher.
