# Supplementary material for: Predicting the risk of HIV infection among internal migrant MSM in China: An optimal model based on three variable selection methods
Source: Front Public Health. 2022 Oct 25;10:1015699. doi: 10.3389/fpubh.2022.1015699 (PMC9641070; doi:10.3389/fpubh.2022.1015699)
Supplement: Supplementary file 1 [file Table_1.DOCX]

Supplementary Table 1. Random forest variable screening results.

| variables | Median Imp | Min Imp | Max Imp | Norm Hits | Decision |
| --- | --- | --- | --- | --- | --- |
| Age | 0.888 | -1.580 | 3.983 | 0.101 | Rejected |
| Education | 5.105 | 2.327 | 8.080 | 0.919 | Confirmed |
| Marriage | 1.566 | -0.389 | 4.013 | 0.101 | Rejected |
| Monthly income | 1.366 | -1.021 | 3.262 | 0.020 | Rejected |
| Time for residence | 0.611 | -0.789 | 2.340 | 0.010 | Rejected |
| Sexual orientation | 1.247 | -0.502 | 2.806 | 0.040 | Rejected |
| HIV Education | -0.991 | -2.999 | 2.094 | 0.000 | Rejected |
| VCT | -0.195 | -1.493 | 1.397 | 0.000 | Rejected |
| Smoking | -0.192 | -2.153 | 1.580 | 0.000 | Rejected |
| Verbal Violence | 0.075 | -1.942 | 2.560 | 0.020 | Rejected |
| Physical violence | -0.032 | -2.414 | 2.136 | 0.000 | Rejected |
| Regular homosexual anal sex partners | -0.249 | -2.355 | 1.544 | 0.000 | Rejected |
| Irregular homosexual anal sex partners | -0.005 | -0.794 | 0.868 | 0.000 | Rejected |
| Homosexual sex trade | -0.370 | -2.232 | 1.832 | 0.000 | Rejected |
| Drinking before sex | 0.635 | -0.956 | 1.654 | 0.000 | Rejected |
| Substance use | 2.238 | -1.273 | 4.541 | 0.384 | Rejected |
| PrEP | -0.438 | -2.598 | 1.729 | 0.000 | Rejected |
| Syphilis | 2.725 | -0.697 | 5.256 | 0.606 | Rejected |
| Score of CUAS | 3.474 | 0.905 | 6.668 | 0.768 | Confirmed |
| Score of CUSS | 4.117 | 1.268 | 6.755 | 0.798 | Confirmed |
| Score of CUSNSS | 0.889 | -1.329 | 4.016 | 0.040 | Rejected |
| Score of CUSES | 3.083 | 0.017 | 5.562 | 0.626 | Rejected |
| Score of RSES | 4.497 | 1.722 | 7.498 | 0.899 | Confirmed |
| Score of ULS | 4.307 | 1.016 | 7.471 | 0.889 | Confirmed |
| Score of PHQ-9 | 3.341 | 0.384 | 5.484 | 0.737 | Confirmed |
| Score of ES | 6.872 | 3.146 | 10.049 | 0.990 | Confirmed |
| Score of DS | 7.618 | 5.128 | 11.438 | 0.990 | Confirmed |
| Score of INQ-15 | 2.799 | -0.338 | 5.229 | 0.556 | Confirmed |
| Score of SAS | 1.271 | -1.777 | 2.993 | 0.020 | Rejected |
| Score of SSS | 4.485 | 1.904 | 6.640 | 0.879 | Confirmed |
| Score of SCS | 2.511 | -0.048 | 5.197 | 0.485 | Confirmed |
