# Supplementary material for: Predicting the risk of HIV infection among internal migrant MSM in China: An optimal model based on three variable selection methods
Source: Front Public Health. 2022 Oct 25;10:1015699. doi: 10.3389/fpubh.2022.1015699 (PMC9641070; doi:10.3389/fpubh.2022.1015699)
Supplement: Supplementary file 2 [file Table_2.DOCX]

Supplementary Table 2. Stepwise regression selection process of model B

|  | Variables | DF | Deviance | AIC |
| --- | --- | --- | --- | --- |
| + | Score of CUSNSS | 1 | 464.23 | 500.23 |
| + | Score of INQ-15 | 1 | 464.27 | 500.27 |
| - | Score of ES | 1 | 468.34 | 500.34 |
| + | Score of SCS | 1 | 464.51 | 500.51 |
| + | Irregular homosexual anal sex partners | 1 | 464.58 | 500.58 |
| - | Score of ULS | 1 | 468.64 | 500.64 |
| - | Syphilis | 1 | 468.69 | 500.69 |
| + | Sexual orientation | 1 | 464.71 | 500.71 |
| + | Score of CUSES | 1 | 464.77 | 500.77 |
| + | Score of SSS | 1 | 464.79 | 500.79 |
| + | Physical violence | 1 | 464.85 | 500.85 |
| + | HIV Education | 1 | 464.87 | 500.87 |
| + | Regular homosexual anal sex partners | 1 | 464.89 | 500.89 |
| + | VCT | 1 | 464.93 | 500.93 |
| + | PrEP | 1 | 464.94 | 500.94 |
| - | Marriage | 2 | 471.17 | 501.17 |
| - | Score of DS | 1 | 469.22 | 501.22 |
| - | Education | 3 | 473.54 | 501.54 |
| + | Time for residence | 2 | 463.56 | 501.56 |
| - | Score of CUSS | 1 | 471.68 | 503.68 |
| + | Smoking | 3 | 463.79 | 503.79 |
| + | Drinking before sex | 3 | 464.73 | 504.73 |
| - | Monthly income | 3 | 477.05 | 505.05 |

Note: the AIC of model B was 498.94.
