# Supplementary material for: Predicting the risk of HIV infection among internal migrant MSM in China: An optimal model based on three variable selection methods
Source: Front Public Health. 2022 Oct 25;10:1015699. doi: 10.3389/fpubh.2022.1015699 (PMC9641070; doi:10.3389/fpubh.2022.1015699)
Supplement: Supplementary file 3 [file Table_3.DOCX]

Supplementary Table 3. Results of univariate logistic regression.

| Variables | Estimate | Std. Error | Z value | *P* value | OR (95%CI) |
| --- | --- | --- | --- | --- | --- |
| Score of CUAS | -0.094 | 0.029 | -3.262 | 0.001 | 0.910 (0.860 - 0.963) |
| Score of CUSS | -0.067 | 0.018 | -3.66 | < 0.001 | 0.935 (0.902 - 0.970) |
| Score of CUSNSS | -0.051 | 0.031 | -1.671 | 0.095 | 0.950 (0.896 - 1.010) |
| Score of CUSES | -0.043 | 0.021 | -2.100 | 0.036 | 0.958 (0.921 - 0.998) |
| Score of ULS | -0.040 | 0.023 | -1.761 | 0.078 | 0.961 (0.919 - 1.004) |
| Substance use | -0.474 | 0.231 | -2.056 | 0.040 | 0.622 (0.394 - 0.975) |
| Syphilis | 0.835 | 0.339 | 2.461 | 0.014 | 2.306 (1.145 - 4.378) |
| Education |  |  |  |  |  |
| Primary school and below | - | - | - | - | 1 |
| Junior high school | -0.652 | 0.667 | -0.978 | 0.328 | 0.521 (0.145 - 2.120) |
| High school | -1.076 | 0.667 | -1.613 | 0.107 | 0.341 (0.095 - 1.388) |
| College and above | -1.863 | 0.650 | -2.864 | 0.004 | 0.155 (0.045 - 0.615) |
| Monthly income ($) |  |  |  |  |  |
| ≤446.40 | - | - | - | - | 1 |
| 446.55-892.80 | 0.997 | 0.315 | 3.168 | 0.002 | 2.711 (1.495 - 5.178) |
| 892.95-1785.60 | 0.290 | 0.355 | 0.816 | 0.414 | 1.336 (0.670 - 2.724) |
| ≥1785.75 | -0.363 | 0.534 | -0.679 | 0.497 | 0.696 (0.220 - 1.867) |
